# Prognostic evaluation in gallbladder carcinoma: Introducing a composite risk model integrating nutritional and immune markers

**DOI:** 10.17305/bb.2024.10673

**Published:** 2024-07-26

**Authors:** Si-qi Yang, Rui-qi Zou, Yu-shi Dai, Hai-jie Hu, Fu-yu Li

**Affiliations:** 1 Division of Biliary Tract Surgery, Department of General Surgery, West China Hospital, Sichuan University, Chengdu, Sichuan Province, China

**Keywords:** Gallbladder carcinoma (GBC), prognostic nutritional index (PNI), glucose-to-lymphocyte ratio (GLR), risk model, curative-intent surgery, prognosis

## Abstract

The importance of evaluating the nutritional status and immune condition prior to surgery has gained significant attention in predicting the prognosis of cancer patients in recent years. The objective of this study is to establish a risk model for predicting the prognosis of gallbladder carcinoma (GBC) patients. Data from GBC patients who underwent radical resection at West China Hospital of Sichuan University (China) from 2014 to 2021 were retrospectively collected. A novel risk model was created by incorporating the prognostic nutritional index (PNI) and glucose-to-lymphocyte ratio (GLR), and each patient was assigned a risk score. The patients were then divided into low- and high-risk cohorts, and comparisons were made between the two groups in terms of clinicopathological features and prognosis. Propensity score matching (PSM) was conducted to reduce potential bias. A total of 300 GBC patients receiving radical surgery were identified and included in this study. Patients in the high-risk group were older, had higher levels of serum carcinoembryonic antigen (CEA), cancer antigen 125 (CA125), and cancer antigen 19-9 (CA19-9), were more likely to experience postoperative complications, and had more aggressive tumor characteristics, such as poor differentiation, lymph node metastasis, and advanced tumor stage. They also had lower overall survival (OS) rates (5-year OS rate: 11.2% vs 37.4%) and disease-free survival (DFS) rates (5-year DFS rate: 5.1% vs 18.2%). After PSM, the high-risk population still experienced poorer prognosis (5-year OS rate: 12.7% vs 20.5%; 5-year DFS rate: 3.2% vs 8.2%). The risk model combining PNI and GLR can serve as a standalone predictor for the prognosis and assist in optimizing the treatment approach for GBC patients.

## Introduction

Gallbladder carcinoma (GBC) is the most common tumor within the biliary system and ranks fifth in frequency among digestive tract tumors. Despite this, its global incidence is relatively low, with significant regional variations [1]. In Western countries like the United States, the incidence is reported at 8.5 cases per 100,000 individuals, while regions, such as Chile and Northern India report higher rates of 27 and 21.5 cases per 100,000, respectively [2, 3]. Known risk factors for GBC include gender, age, and the presence of gallbladder stones or polyps [3]. The lack of reliable screenings, coupled with the early onset of subtle symptoms and the cancer’s rapid spread, often leads to delayed diagnosis and poor prognosis in most GBC cases [4]. Currently, the majority of GBC cases are incidentally discovered during surgery or postoperative analysis of cholecystectomy procedures performed for non-cancerous gallbladder conditions. The reported prevalence of incidentally discovered GBC ranges from 0.14% to 1.6% [5–7]. Surgery remains the only treatment option for GBC, and with advancements in surgical techniques and postoperative care, the current 5-year survival rate ranges from 22% to 38% [8–10].

Prognostic indicators for GBC include pathologic parameters, such as the AJCC eighth edition TNM staging system, tumor differentiation, and tumor necrosis [11–13]. However, these parameters are often difficult to obtain preoperatively, as they require surgical resection samples. This challenge complicates risk stratification and identifying high-risk patients who may benefit from more aggressive treatments. Several studies have explored the significance of preoperative inflammatory and nutritional status in predicting the prognosis of GBC patients. Inflammatory markers, including the neutrophil-to-lymphocyte ratio (NLR), platelet-to-lymphocyte ratio (PLR), and lymphocyte-to-monocyte ratio (LMR), have shown correlations with prognosis [14–17]. Preoperative nutritional indicators, such as the prognostic nutritional index (PNI) and controlling nutritional status (CONUT), have also been linked to survival outcomes in cancer patients [18–20]. Additionally, the preoperative glucose-to-lymphocyte ratio (GLR) has been identified as a sensitive indicator for evaluating glucose metabolism, cancer aggressiveness, and immunological status in various cancers, including hepatocellular carcinoma, gastric cancer, and T2 stage GBC [21–23].

Most prognostic models developed for GBC have relied on tumor markers or pathological parameters. For instance, Chen et al. [24] assessed the prognostic significance of the systemic immune inflammation index in GBC. A recent study developed a predictive model for long-term survival in GBC based on cancer antigen 19–9 (CA19-9), peripheral organ invasion, lymph node status, and tumor location [25]. However, relying solely on a single factor often overlooks tumor biology and individual patient characteristics, such as nutritional status or immune function. Additionally, the variability in case selection criteria and laboratory standards across different prognostic models limits their clinical utility [26, 27]. In this study, we established an innovative risk model incorporating GLR and PNI to preoperatively stratify patients with GBC and predict their prognosis.

## Materials and methods

### Patient selection

We retrospectively compiled the medical data of patients diagnosed with GBC who underwent radical resection at West China Hospital of Sichuan University, China, from January 2014 to December 2021. The dataset included demographic details, laboratory test results, surgical information, and pathological diagnosis reports. To be included, patients had to meet the following criteria: (1) confirmed GBC diagnosis according to the WHO’s 2019 classification, (2) complete clinical and follow-up data (patients with sufficient survival data for a recorded survival period > 0 months), (3) absence of diabetes, and (4) achievement of R0 resection.

### Follow-up assessments

All patients were regularly monitored through telephone interviews or outpatient examinations. During the first postoperative year, follow-up assessments were conducted every three months and then every six months thereafter. These assessments included physical examinations, liver function tests, serum levels of CA19-9 and CEA, and computed tomography (CT) or magnetic resonance imaging (MRI) of the chest and abdomen. Overall survival (OS) was defined as the time from the date of radical surgery to either the date of death from any cause or the most recent follow-up date. Disease-free survival (DFS) was calculated from the date of surgery to the most recent follow-up date unless there was a recurrence during the follow-up period. The most recent follow-up was completed in December 2023.

### Data collection

Data on age, sex, BMI, preoperative lymphocyte count, preoperative blood glucose, and preoperative levels of serum CA19-9, cancer antigen 125 (CA125), CEA, and albumin were obtained from medical records. Observations with missing data were excluded from the analysis. GLR and PNI were calculated using the following formulas: GLR ═ preoperative blood glucose (mmol/L)/total lymphocyte count (*109/L); PNI ═ albumin level (g/L) + 5 × total lymphocyte count (*109/L). Tumor features, such as liver resection, bile duct resection, subtypes, differentiation, perineural invasion, lymph node metastasis, T stage, and postoperative complications (Clavien–Dindo grade ≥ II), were determined based on intraoperative data and postoperative pathological results. The data collection table can be found in the supplementary materials.

### Construction of the risk model

Univariate and multivariate Cox regression analyses were applied to identify the associations between GLR, PNI, and the survival of GBC patients to build the risk model. Using the “survival” R package, the risk score for each patient was calculated using the following formula: risk score ═ PNI*β1+ GLR*β2 (The R script is available in the supplementary materials). Receiver operating characteristic (ROC) curve analysis was used to determine the optimal cutoff value for the risk score. Based on this value, patients were classified into low- and high-risk populations.

### Ethical statement

Approval for this study was granted by the Institutional Ethics Review Board of West China Hospital, and the requirement for informed consent was waived due to the retrospective nature of the study.

### Statistical analysis

IBM SPSS 23.0 (Chicago, IL, USA), GraphPad Prism 8, and R statistical software (v4.2.1) were used to conduct statistical analysis. Median values and ranges were used to summarize continuous variables, while categorical variables were presented as absolute numbers and percentages. Group comparisons were made using appropriate tests such as Fisher’s exact test, chi-squared test, or Mann–Whitney *U* test. The Kaplan–Meier method, along with log-rank tests, was utilized to estimate survival probabilities. The independent prognostic value of factors was evaluated by univariate and multivariate Cox regression analyses. To reduce confounding bias, propensity score matching (PSM) analysis was conducted based on age, serum levels of CEA, CA125, and CA19-9, postoperative complications, tumor differentiation, node metastasis, and tumor stage. Low-risk controls were matched to high-risk cases at a 1:1 ratio using the closest matched propensity score and a caliper width of 0.02 standard deviations. A two-tailed *P* value < 0.05 was considered statistically significant.

**Table 1:** Clinical features of all included patients

**Variables**	**All (*n* ═ 300)**	**High risk (*n* ═ 150, 50.0%)**	**Low risk (*n* ═ 150, 50.0%)**	***P* value**
Age (years)				0.008
≤60	129 (43.0%)	57 (38.0%)	72 (48.0%)	
>60	171 (57.0%)	93 (62.0%)	78 (52.0%)	
Sex				0.724
Male	121 (40.3%)	59 (39.3%)	62 (51.7%)	
Female	179 (59.7%)	91 (60.7%)	88 (73.3%)	
BMI (kg/m^2^)				0.166
≤23	144 (48.0%)	66 (44.0%)	78 (52.0%)	
>23	156 (52.0%)	84 (56.0%)	72 (48.0%)	
CEA (ng/mL)				0.01
≤5	230 (76.7%)	103 (68.7%)	127 (80.0%)	
>5	70 (23.3%)	47 (31.3%)	23 (20.0%)	
CA125 (U/mL)				0.022
≤24	193 (64.3%)	87 (58.0%)	106 (70.7%)	
>24	107 (35.7%)	63 (42.0%)	44 (29.3%)	
CA19-9 (U/mL)				0.028
≤30	149 (41.3%)	65 (38.0%)	84 (44.7%)	
>30	151 (58.7%)	85 (62.0%)	66 (55.3%)	
Gallbladder stones				0.908
Present	145 (48.3%)	72 (48.0%)	73 (48.7%)	
Absent	155 (51.7%)	78 (52.0%)	77 (51.3%)	
Liver resection				0.465
Yes	198 (66.0%)	102 (68.0%)	96 (64.0%)	
No	102 (34.0%)	48 (32.0%)	54 (36.0%)	
Bile duct resection				0.133
Yes	145 (48.3%)	79 (52.7%)	66 (44.0%)	
No	155 (51.7%)	71 (47.3%)	84 (56.0%)	
Postoperative complication				0.012
Present	77 (25.6%)	48 (32.0%)	29 (19.3%)	
Absent	223 (74.3%)	102 (68.0%)	121 (80.7%)	
Pathology				0.197
Adenocarcinoma	267 (89.0%)	130 (86.7%)	137 (91.3%)	
Others	33 (11.0%)	20 (13.3%)	13 (8.7%)	
Differentiation				0.049
Poor	139 (46.3%)	78 (52.0%)	61 (40.7%)	
Moderate/Well	161 (53.7%)	72 (48.0%)	89 (59.3%)	
Perineural invasion				0.435
Present	49 (16.3%)	27 (18.0%)	22 (14.7%)	
Absent	101 (83.7%)	123 (82.0%)	128 (85.3%)	
Node metastasis				0.003
Yes	117 (39.0%)	71 (47.4%)	46 (30.7%)	
No	183 (61.0%)	79 (52.6%)	104 (69.3%)	
pT (8th AJCC)				0.031
T1/T2	228 (76.0%)	106 (70.7%)	122 (81.3%)	
T3	72 (24.0%)	44 (29.3%)	28 (18.7%)	

AJCC: American Joint Committee on Cancer; BMI: Body mass index; CA125: Carbohydrate antigen 125; CA19-9: Carbohydrate antigen 19-9; CEA: Carcinoembryonic antigen.

**Table 2:** Univariate and multivariate analyses of overall survival and disease-free survival

**Variables**	**Univariate analysis**			**Multi variate analysis**		
	**HR**	**95% CI**	***P* value**	**HR**	**95% CI**	***P* value**
*Overall survival*						
Age (<60 vs ≥60)	2.439	1.822–3.266	<0.0 01	/	/	0.182
Sex (male vs female)	/	/	0.713	/	/	/
BMI (≤23 vs >23)	/	/	0.476	/	/	/
CEA (≤5 vs >5)	/	/	0.872	/	/	/
CA125 (>24 vs ≤24)	/	/	0.61	/	/	/
CA19-9 (>30 vs ≤30)	/	/	0.723	/	/	/
GLR	1.506	1.136–1.996	0.004	1.811	1.330–3.440	<0.0 01
PNI	2.639	1.962–3.549	<0.0 01	2.320	1.552–3.368	<0.0 01
Gallbladder stones	/	/	0.728	/	/	/
Liver resection	/	/	0.184	/	/	/
Bile duct resection	/	/	0.12	/	/	/
Postoperative complication	/	/	0.415	/	/	/
Pathology (adenocarcinoma vs other)	/	/	0.549	/	/	/
Differentiation (poor vs moderate/well)	1.613	1.217–2.138	0.001	1.359	1.011–1.827	0.420
Perineural invasion (positive vs negative)	1.561	1.091–2.234	0.015	/	/	0.606
Node metastasis (positive vs negative)	2.483	1.857–3.322	<0.001	1.778	1.320–2.397	<0.001
pT (8th AJCC) (T1/T2 vs T3)	2.412	1.917–3.035	<0.001	1.816	1.423–2.317	<0.001
*Disease-free survival*						
Age (<60 vs ≥60)	2.076	1.581–2.726	<0.0 01	/	/	0.356
Sex (male vs female)	/	/	0.702	/	/	/
BMI (≤23 vs >23)	/	/	0.994	/	/	/
CEA (≤5 vs >5)	/	/	0.592	/	/	/
CA125 (>24 vs ≤24)	/	/	0.846	/	/	/
CA19-9 (>30 vs ≤30)	/	/	0.92	/	/	/
GLR	1.663	1.273–2.173	<0.0 01	1.872	1.403–2.497	<0.0 01
PNI	2.314	1.755–3.052	<0.0 01	2.225	1.528–3.241	<0.0 01
Gallbladder stones	/	/	0.808	/	/	/
Liver resection	/	/	0.325	/	/	/
Bile duct resection	/	/	0.18	/	/	/
Postoperative complication	/	/	0.12	/	/	
Pathology (adenocarcinoma vs other)	/	/	0.606	/	/	
Differentiation (poor vs moderate/well)	1.405	1.075–1.838	0.013	/	/	0.234
Perineural invasion (positive vs negative)	/	/	0.06	/	/	/
Node metastasis (positive vs negative)	2.25	1.715–2.952	<0.001	1.824	1.380–2.412	<0.001
pT (8th AJCC) (T1/T2 vs T3)	1.931	1.265–2.382	<0.001	1.486	1.19–1.857	<0.001

AJCC: American Joint Committee on Cancer; BMI: Body mass index; CA125: Carbohydrate antigen 125; CA19-9: Carbohydrate antigen 19-9; CEA: Carcinoembryonic antigen; GLR: Glucose-to-lymphocyte ratio; PNI: Prognostic nutritional index.

## Results

### Patient characteristics

We identified 401 patients with pathologically confirmed GBC through database searches. Of these, 101 patients were excluded from the study: 21 due to R1 resection, 46 due to diabetes, and 34 due to missing clinical and follow-up data. Ultimately, 300 eligible patients were included in our study. Table 1 provides the clinicopathological features of all participants. Based on the defined risk score cutoff value, we categorized these patients into low- and high-risk populations. There were no significant differences between the two cohorts regarding sex ratio, BMI, preoperative gallbladder stones, liver resection, choledochotomy, pathology subtype, or the presence of perineural invasion. However, high-risk patients exhibited elevated levels of serum CEA, CA125, and CA19-9, a higher frequency of postoperative complications, and more aggressive tumor features, such as poor differentiation, node metastasis, and advanced tumor stage.

### Construction and cutoff value of the risk score

**Figure 1.**
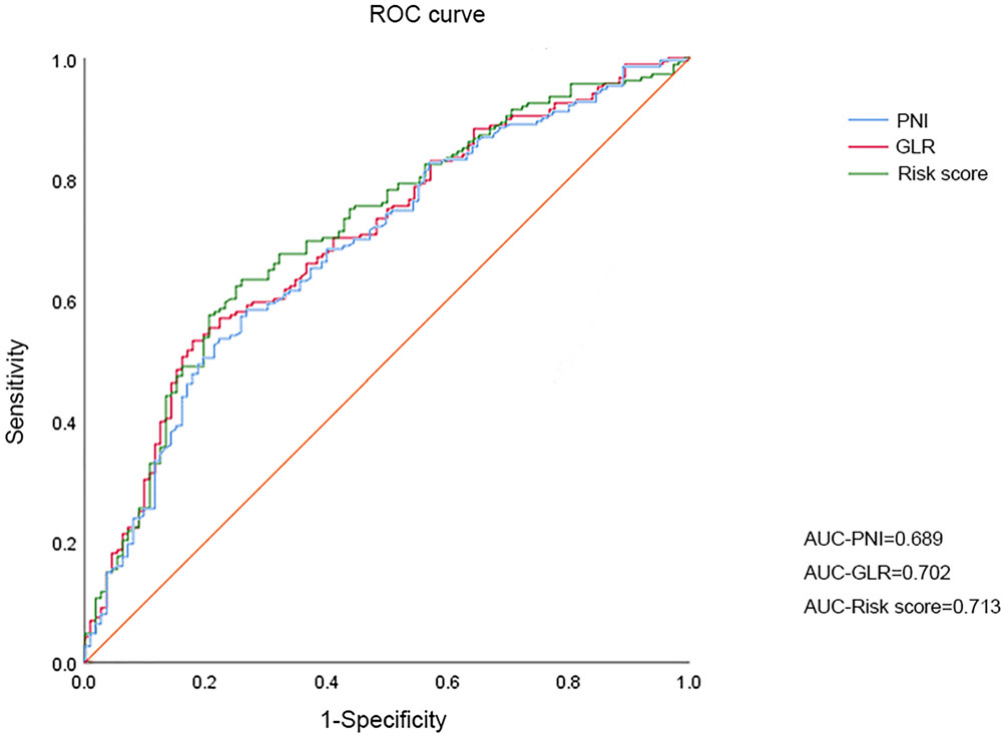
**Analysis of the ROC curve for predicting OS with the risk score, PNI, and GLR.** OS: Overall survival; PNI: Prognostic nutritional index; GLR: Glucose-to-lymphocyte ratio; ROC: Receiver operating characteristic; AUC: Area under the curve.

Through univariate and multivariate Cox regression analyses, we determined the prognostic significance of GLR (Multivariate Cox: OS, HR: 1.811, 95% CI 1.330–3.440; DFS, HR: 1.872, 95% CI 1.403–2.497) and PNI (Multivariate Cox: OS, HR: 2.320, 95% CI 1.552–3.368; DFS, HR: 2.225, 95% CI 1.403–2.497), as detailed in Table 2. A risk score was calculated for each GBC patient using the formula: risk score ═ GLR*0.012 - PNI*0.07. The area under the curve (AUC) for GLR, PNI, and risk score was determined through ROC curve analysis, with the risk score having the highest AUC (0.713) compared to GLR (0.702) and PNI (0.689). Further ROC analyses were performed for T stage (AUC ═ 0.695) and node metastasis (AUC ═ 0.620), indicating the superior predictive ability of the risk model (Figure 1). The optimal cutoff value for the risk score was identified as 1.27.

### Prognostic significance of risk model

**Figure 2.**
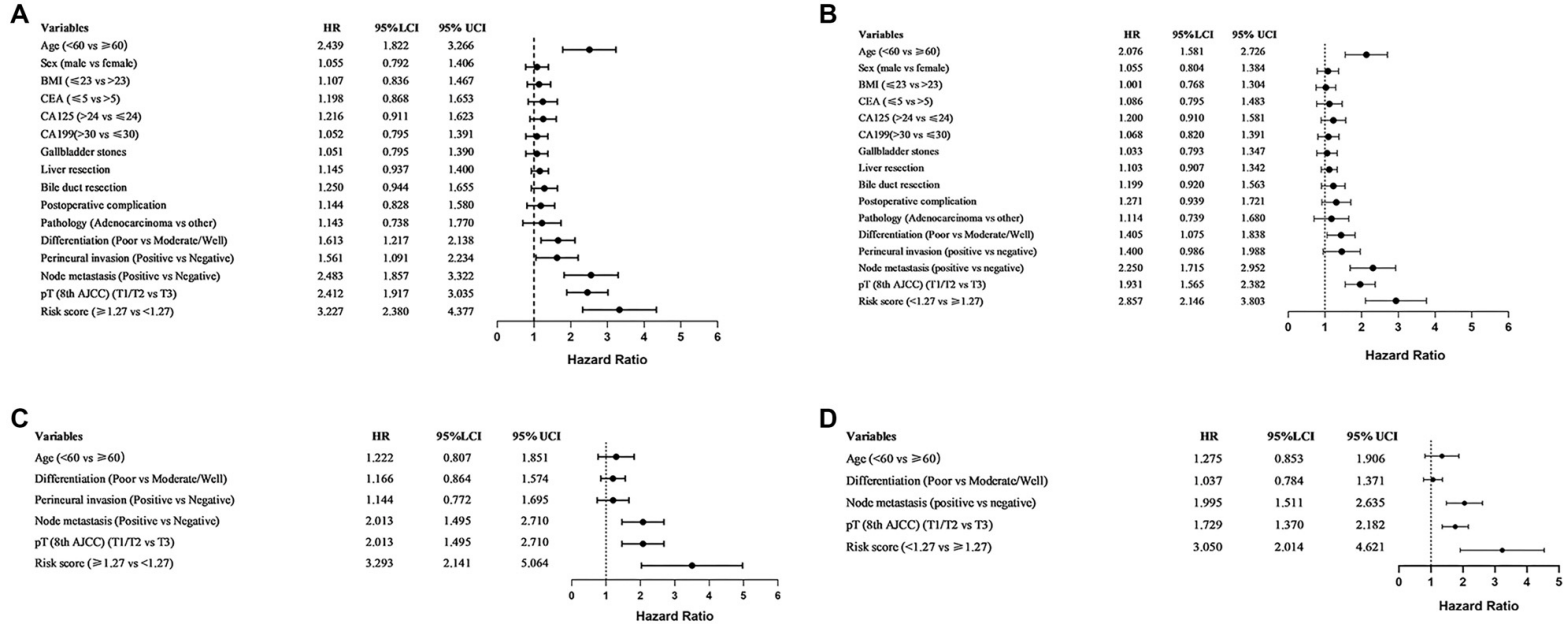
**Cox-regression analysis for OS and DFS.** (A) Univariate analysis for overall survival; (B) Multivariate analysis for overall survival; (C) Univariate analysis for disease-free survival; (D) Multivariate analysis for disease-free survival. OS: Overall survival; DFS: Disease-free survival.

To investigate the prognostic significance of our risk model, we performed a Cox regression analysis. Univariate analysis revealed that age (HR 2.439, 95% CI 1.822–3.266), tumor differentiation (HR 1.613, 95% CI 1.217–2.138), perineural invasion (HR 1.561, 95% CI 1.091–2.234), node metastasis (HR 2.483, 95% CI 1.857–3.322), T stage (HR 2.412, 95% CI 1.917–3.035), and risk score (HR 3.227, 95% CI 2.380–4.377) were prognostic factors for OS (Figure 2A). Subsequent multivariate analysis identified node metastasis (HR 2.013, 95% CI 1.495–2.710), T stage (HR 2.013, 95% CI 1.495–2.710), and risk score (HR 3.293, 95% CI 2.141–5.064) as independent prognostic factors for OS (Figure 2B). Regarding DFS, univariate analysis demonstrated associations between age (HR 2.076, 95% CI 1.581–2.726), tumor differentiation (HR 1.405, 95% CI 1.075–1.838), node metastasis (HR 2.250, 95% CI 1.715–2.952), T stage (HR 1.931, 95% CI 1.565–2.382), and risk score (HR 2.857, 95% CI 2.146–3.803) (Figure 2C). Multivariate analysis further highlighted node metastasis (HR 1.996, 95% CI 1.495–2.710), T stage (HR 1.729, 95% CI 1.370–2.182), and risk score (HR 3.050, 95% CI 2.014–4.621) as independent factors for DFS (Figure 2D).

### Survival outcomes

**Figure 3.**
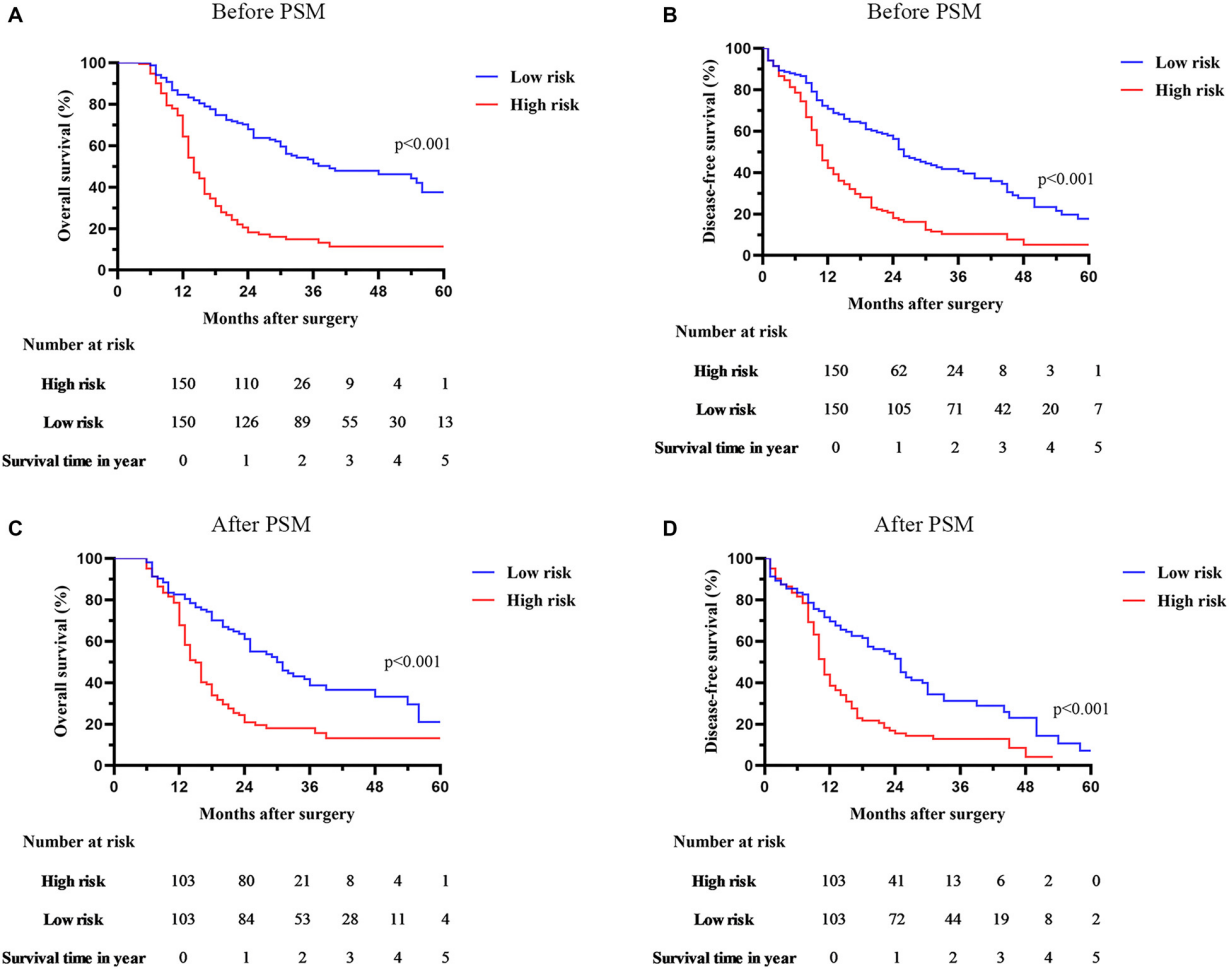
**Comparison of survival outcomes between low- and high-risk cohort.** (A) Overall survival before PSM; (B) Disease-free survival before PSM; (C) Overall survival after PSM; (D) Disease-free survival after PSM. PSM: Propensity score matching.

We compared the survival outcomes of patients with different risk scores, specifically examining OS and DFS. According to the Kaplan–Meier survival curves (Figure 3A and 3B), high-risk patients had poorer OS and DFS. In the low-risk group, the 1-, 3-, and 5-year OS rates were 85.0%, 53.1%, and 37.4%, respectively. In the high-risk group, these rates were 74.5%, 15.2%, and 11.2% at 1, 3, and 5 years, respectively. Furthermore, the high-risk group had 1-, 3-, and 5-year DFS rates of 66.3%, 29.1%, and 13.4%, respectively.

PSM analysis was used to address selection bias between individuals with different risk scores. A 1:1 PSM process was employed, considering factors, such as age, serum CEA, CA125, CA19-9, postoperative complications, tumor differentiation, node metastasis, and tumor stage. As a result, the two cohorts were effectively balanced, and there were no significant differences in clinicopathological features (Table 3). However, despite this balance, patients with high-risk scores still had lower OS and DFS rates compared to those with low-risk scores (Figure 3C and 3D). The low-risk group had 1-, 3-, and 5-year OS rates of 82.4%, 41.3%, and 20.5%, respectively, while the high-risk group had rates of 78.5%, 70.6%, and 12.7% at 1, 3, and 5 years, respectively. Similarly, the low-risk group had 1-, 3-, and 5-year DFS rates of 71.6%, 30.6%, and 8.2%, respectively, while the high-risk group had rates of 44.7%, 12.5%, and 3.2%.

**Table 3:** Clinical features of patients with different risk after PSM

**Variables**	**Low risk (*n* ═ 103)**	**High risk (*n* ═ 103)**	***P* value**
Age			0.780
≤60	48 (32.0%)	46 (44.7%)	
>60	55 (68.0%)	57 (55.3%)	
Sex			0.203
Male	38 (36.2%)	46 (44.7%)	
Female	65 (63.1%)	57 (553%)	
BMI (kg/m^2^)			0.676
≤23	50 (48.5%)	53 (51.5%)	
>23	53 (51.5%)	50 (48.5%)	
CEA (ng/mL)			0.503
≤5	82 (79.6%)	78 (75.7%)	
>5	21 (20.4%)	25 (24.3%)	
CA125 (U/mL)			0.236
≤24	73 (70.9%)	65 (63.1%)	
>24	30 (29.1%)	38 (36.9%)	
CA19-9 (U/mL)			0.889
≤30	52 (50.5%)	53 (51.5%)	
>30	51 (49.5%)	50 (48.5%)	
Gallbladder stones			0.329
Present	56 (54.4%)	49 (47.6%)	
Absent	47 (45.6%)	54 (52.4%)	
Liver resection			0.769
Yes	67 (65.0%)	69 (67.0%)	
No	36 (35.0%)	34 (33.0%)	
Bile duct resection			0.329
Yes	46 (44.7%)	53 (51.5%)	
No	57 (55.3%)	50 (48.5%)	
Postoperative complication			0.870
Present	25 (24.3%)	24 (23.3%)	
Absent	78 (75.7%)	79 (76.7%)	
Pathology			0.250
Adenocarcinoma	95 (92.2%)	90 (87.4%)	
Others	8 (7.8%)	13 (13.6%)	
Differentiation			0.889
Poor	50 (48.5%)	52 (50.5%)	
Moderate/Well	53 (51.5%)	51 (49.5%)	
Perineural invasion			0.856
Present	19 (18.4%)	18 (17.5%)	
Absent	84 (81.6%)	85 (82.5%)	
Node metastasis			0.666
Yes	40 (38.8%)	37 (35.9%)	
No	63 (61.2%)	66 (64.1%)	
pT (8th AJCC)			0.330
T1/T2	75 (72.8%)	81 (78.6%)	
T3	28 (27.2%)	22 (21.4%)	

AJCC: American Joint Committee on Cancer; BMI: Body mass index; CA125: Carbohydrate antigen 125; CA19-9: Carbohydrate antigen 19-9; CEA: Carcinoembryonic antigen; PSM: Propensity score matching.

**Figure 4.**
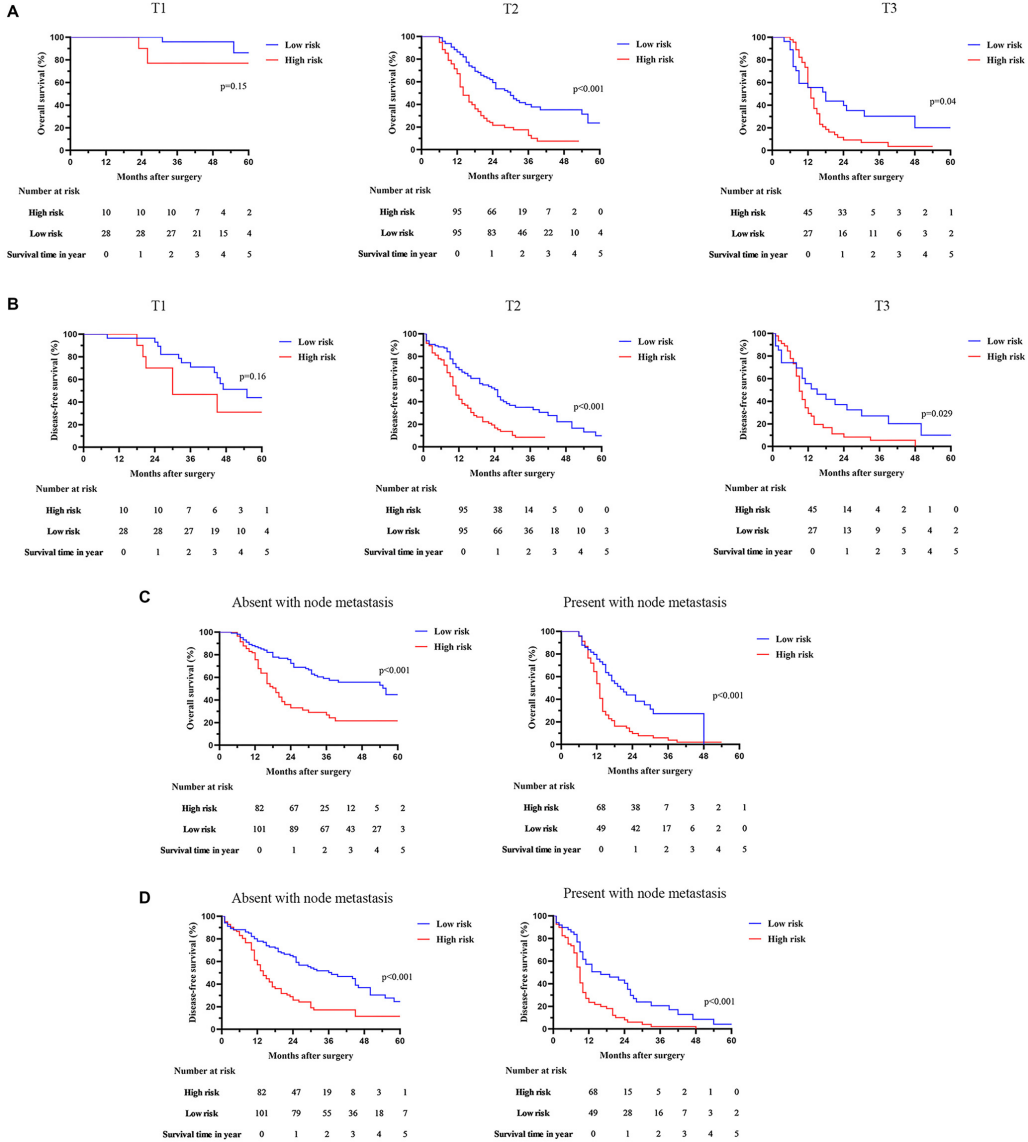
**Associations of risk score with the survival outcome of GBC patients stratified based on the T stage.** (A) Overall survival; (B) Disease-free survival, and node metastasis; (C) Overall survival; (D) Disease-free survival. GBC: Gallbladder carcinoma.

Furthermore, we examined the relationship between risk score and survival in patients with GBC, stratifying them by T stage and node metastasis. For T1 GBC, the OS (*P* ═ 0.15) and DFS (*P* ═ 0.16) were comparable between low- and high-risk cohorts (Figure 4A and 4B). However, for T2-3 GBC, patients in the low-risk group experienced significantly better OS and DFS (Figure 4A and 4B). Thus, our results indicate that the calculated risk score effectively predicts the prognosis of patients with T2-3 GBC. Moreover, patients with higher risk scores consistently showed lower OS and DFS even when considering their lymph node status (Figure 4C and 4D). These findings underscore the accuracy of our risk model in predicting outcomes for GBC patients.

## Discussion

In recent years, there has been significant interest in the role of preoperative nutritional assessment and immune status in predicting outcomes for cancer patients. PNI, first introduced by Buzby et al. [28], is a recognized indicator of both nutritional and inflammatory conditions. Studies have consistently demonstrated a link between PNI and poor prognosis in individuals with gastric, esophageal, and breast cancer [29–32]. Additionally, multiple studies have independently verified the prognostic value of PNI in patients with biliary tract tumors [33].

Tumor cells exhibit higher metabolic activity than normal cells, necessitating increased glucose consumption. This phenomenon is exploited in oncologic imaging using 18F-fluorodeoxyglucose positron emission tomography, which can estimate both tumor glucose metabolism and biological properties [34]. Elevated blood glucose levels are associated with a poorer prognosis in cancer patients and are a significant risk factor for mortality in gastric, lung, and liver cancers [35]. Moreover, high glucose levels are linked to recurrence and metastasis in breast cancer [36]. Cellular studies confirm that a high-glucose environment promotes tumor cell proliferation, activates pro-cancer signaling pathways, and inhibits apoptosis [37, 38].

Lymphocytes play a crucial role in the systemic inflammatory response and are vital for cell-mediated anti-tumor immunity, offering valuable insights into immune system status. Numerous studies have established a strong link between immune status and cancer prognosis. For example, Garnelo et al. [39] found that lower lymphocyte levels were associated with more advanced tumor stages. Similarly, research suggests that the local immune status within tumors can significantly impact the prognosis of patients with biliary tract cancer (BTC), likely due to the beneficial effects of tumor-infiltrating lymphocytes in combating cancer [40]. Conversely, low lymphocyte counts can lead to inadequate immune responses within the tumor microenvironment, promoting cancer progression [41].

Hypoalbuminemia, a deficiency in albumin, has been associated with various dysfunctions, including abnormal activation of systemic inflammation, reduced drug response, and compromised immune function [42]. In individuals with advanced tumors, declining albumin levels may result from factors, such as poor nutritional status, ongoing inflammation, and disease progression, contributing to an unfavorable prognosis [43]. Additionally, it is worth investigating whether there is a connection between elevated blood glucose levels and compromised nutritional and immune status in patients with GBC. Previous studies have shown that preoperative immunonutrition can help regulate inflammatory responses during the perioperative period. However, the specific mechanisms underlying the interaction between high blood glucose levels and nutritional and immune status remain unclear, warranting further research.

In this study, we developed a risk stratification model using preoperative hematologic parameters. Our model integrates lymphocyte counts, blood glucose, and albumin levels to assess patients’ nutritional and inflammatory status more comprehensively. This combined approach showed better predictive power than using GLR or PNI alone (AUCGLR ═ 0.702, AUCPNI ═ 0.689, AUCRisk score ═ 0.713). Based on the risk model, we divided the 300 patients into high- and low-risk populations. Patients in the high-risk group exhibited more aggressive tumor characteristics, including poorer differentiation, higher rates of node metastasis, and more advanced tumors. Survival analysis revealed a significant correlation between a higher risk score and poorer long-term survival and recurrence rates.

To minimize selection bias and balance clinical and pathological differences between high- and low-risk populations, we performed PSM analysis. Following PSM, GBC patients with lower risk scores still demonstrated significantly improved survival outcomes. Subgroup analysis further showed that our risk model had a higher predictive value for T2-3 GBC (*P* < 0.001). Through Cox regression analyses, we identified our risk model as an independent determinant of both OS and DFS for GBC patients. To the best of our knowledge, this research is the first to demonstrate that combining PNI with GLR can provide preoperative risk stratification and prognostic information for GBC patients undergoing radical surgery. These findings underscore the importance of perioperative nutritional support in managing GBC patients undergoing curative-intent surgery. Furthermore, our model can identify patients at risk for poor outcomes preoperatively, which may help guide the selection of aggressive treatment strategies. This risk model offers a practical and cost-effective tool for making informed treatment decisions and improving the prognosis for GBC patients.

Despite our findings, it is important to acknowledge the limitations of our study. First, our study was retrospective, and all patients were sourced from a single center, which may introduce selection bias. Second, our inclusion criteria covered patients who underwent curative-intent surgery with varying operative modalities and substantial differences in the extent of resection, potentially affecting our results. Additionally, although we excluded GBC patients with preoperative diabetes, other factors that can elevate blood glucose levels may have influenced the accuracy of our risk model. Therefore, further high-quality studies with larger sample sizes and prospective or multicenter designs are necessary to confirm the validity of our results.

## Conclusion

Our study concludes that the risk model combining PNI and GLR is an independent predictor of prognosis for GBC patients who have undergone radical surgery. This easily accessible metric can accurately identify GBC patients at risk for poor outcomes prior to surgery, providing invaluable guidance for clinical treatment and improving overall prognosis.

## Supplemental data

**Table S1:** Supplemental data

**ID**	**Time of surgey**	**Age**	**Sex 0: male 1: female**	**Height (cm)**	**Weight (kg)**	**Gall bladder stones 0: absent 1: present**	**Preoperative CA199 (U/mL)**	**Preoperative CA125 (U/mL)**	**Preoperative CEA (ng/mL)**	**Preoperative ALB (g/L)**	**Preoperative total lymphocyte count 10ˆ9/L**	**Preoperative blood sugar (mmol/L)**	**Node metastasis 0: absent 1: present**	**AJCC 8th T stage**	**Differentiation 1: poor 2: moderate 3: high**	**Pathology 0: Adenocarcinoma 1: other**	**Bile duct resection 0: No 1: Yes**	**Liver resection 0: No 1: Yes**	**Perineural invasion 0: No 1: Yes**	**Postoperative complications (Clavien-Dindo grade ≥ II) 0:No 1: Yes**	**Recurrence 0: No 1: Yes**	**Disease-free survival (months)**	**Survival status 0: alive 1: death**	**Overall survival (months)**
1	8/5/2019	52	0	170	73	0	296	32.71	1.01	47	3.47	4.97	2	T3	1	0	1	1	0	0	0	22	0	22
2	2020/11/18	66	1	148	49	0	41.1	29.38	1.93	33	2.72	4.42	0	T3	1	0	1	1	0	0	1	10	0	12
3	2018/11/21	59	0	167	70	0	22.22	5.41	1.27	43.9	2.92	4.84	0	T1b	2	0	0	0	0	0	0	66	0	66
4	4/21/2020	51	1	163	80	1	1.1	15.1	1.63	34.6	2.57	4.34	0	T2a	2	0	0	1	1	0	1	1	1	14
5	11/2/2019	51	0	170	61.5	1	28	21.37	0.27	45.1	2.46	4.17	1	T2a	2	0	1	1	0	0	0	14	0	14
6	7/29/2019	80	1	154	45	1	40.46	19.03	1.76	33.6	2.02	3.64	1	T1b	2	0	0	0	0	0	1	44	0	55
7	2020/12/04	51	0	168	73	0	6.59	55.7	1.06	42.7	2.27	4.12	0	T2a	2	1	1	1	1	0	1	25	0	50
8	6/28/2018	46	0	170	58	0	5.68	16.4	3.31	43.2	2.52	4.82	0	T1b	2	0	0	0	0	0	1	11	1	17
9	7/10/2019	46	1	105	49	0	18.48	13.46	2.21	46.1	2.14	4.24	0	T1a	1	0	0	1	0	0	0	28	0	28
10	5/9/2018	62	0	168	65	0	27	21.28	2.34	44.6	2.32	4.6	1	T1a	2	0	0	1	0	0	0	58	0	58
11	5/21/2020	56	0	160	57	1	13	13.4	1.65	30.1	2.88	5.76	0	T2b	1	0	0	1	1	0	0	27	0	27
12	9/23/2019	50	1	154	53	0	6.5	54.27	3.37	38.7	2.3	4.68	1	T1a	1	1	0	1	0	0	1	30	0	42
13	11/21/2018	47	1	145	49	1	10.83	62.28	2.08	30.9	3.1	6.38	0	T1a	2	0	0	0	0	0	1	23	1	29
14	12/27/2018	69	0	170	62	1	69.14	35.48	1.84	42.4	2.42	5.12	1	T2b	2	0	1	1	0	0	1	26	0	64
15	11/16/2017	62	0	172	78	1	21.84	8.44	1.99	44	1.91	4.13	0	T2a	1	0	0	1	0	0	0	18	0	18
16	11/30/2017	68	0	170	61.5	0	29	18.47	3.46	40.5	2.2	4.76	0	T1b	1	0	0	1	0	0	0	35	0	35
17	12/12/2019	49	0	176	76.5	0	12.1	9.72	1.48	44.3	2.24	4.85	0	T1b	1	0	0	1	0	0	1	19	1	25
18	2018/3/22	41	0	175	62	0	196.8	18.71	1.44	35.3	2.1	4.56	1	T3	2	0	1	0	0	1	1	11	0	12
19	9/6/2018	75	1	148	40	0	36.1	9.3	1.54	36.6	2.58	5.78	2	T2b	2	0	1	0	1	0	1	64	0	26
20	11/1/2018	61	1	158	55	1	41.97	13.5	2.05	29.6	1.98	4.49	0	T2b	2	0	1	1	1	0	1	45	0	60
21	2021/10/25	39	1	155	51	1	11.7	20.3	1.14	39.2	2.1	4.77	0	T2a	2	0	0	1	0	1	0	21	1	21
22	10/12/2020	57	1	161	64	0	1.7	9.08	2.38	45.3	2.37	5.41	0	T2b	1	0	0	0	1	0	0	23	0	23
23	6/19/2019	60	1	159	57	0	8.83	9.34	0.61	42.2	1.99	4.66	0	T2b	1	0	1	0	0	0	1	50	1	56
24	7/9/2020	67	1	155	50.5	1	21	34.9	1.88	44.8	1.82	4.34	0	T2a	1	0	0	1	0	0	1	58	1	64
25	12/11/2019	61	1	163	60	0	70.8	1.27	0.77	38.1	2.3	5.55	0	T1a	2	0	0	1	1	0	1	16	0	32
26	4/26/2020	57	1	158	71	1	10.8	11	1.15	43.7	2.1	5.09	0	T2a	2	0	1	0	0	0	0	36	0	36
27	2021/06/16	67	0	164	61.5	0	25	0.46	1.97	45.3	1.92	4.68	0	T1a	2	0	0	0	0	1	1	1	1	54
28	2021/07/01	56	1	160	73.5	0	1030	10.63	5.7	42	1.81	4.47	1	T2b	2	0	0	0	0	1	0	21	0	21
29	2020/10/26	67	1	160	62.5	1	35.2	35.4	2.88	38	1.78	4.41	0	T2b	2	0	0	1	0	1	0	36	0	36

## Data Availability

All data generated or analyzed during this study is included in the published article.
